# Susceptibility of Virulent *Yersinia pestis* Bacteria to Predator Bacteria in the Lungs of Mice

**DOI:** 10.3390/microorganisms7010002

**Published:** 2018-12-21

**Authors:** Riccardo Russo, Irina Kolesnikova, Thomas Kim, Shilpi Gupta, Androulla Pericleous, Daniel E. Kadouri, Nancy D. Connell

**Affiliations:** 1 Department of Medicine and the Center for Emerging Pathogens, Rutgers, New Jersey Medical School, Newark, NJ 07101, USA; russori@njms.rutgers.edu (R.R.); kolesnir@njms.rutgers.edu (I.K.); tk473@njms.rutgers.edu (T.K.); 2 Department of Oral Biology, Rutgers School of Dental Medicine, Newark, NJ 07101, USA; sg1135@sdm.rutgers.edu (S.G.); andrea83@gsbs.rutgers.edu (A.P.); kadourde@sdm.rutgers.edu (D.E.K.); 3 Center for Health Security, Johns Hopkins Bloomberg School of Public Health, Baltimore, MD 20102, USA

**Keywords:** predatory bacteria, *Bdellovibrio*, *Yersinia pestis*, lung infection, select agent countermeasure

## Abstract

Multi-drug resistant bacterial infections are a serious threat to global public health. Changes in treatment modalities and prudent use of antibiotics can assist in reducing the threat, but new approaches are also required for untreatable cases. The use of predatory bacteria, such as *Bdellovibrio* *bacteriovorus*, is among the novel approaches being considered as possible therapeutics for antibiotic resistant and/or unidentified bacterial infections. Previous studies have examined the feasibility of using predatory bacteria to reduce colony-forming units (CFUs) in the lungs of rats exposed to lethal doses of *Klebsiella pneumoniae*; here we apply the approach to the Tier 1 select agent *Yersinia pestis*, and show that three doses of *B. bacteriovorus* introduced every six hours reduces the number of CFUs of *Y. pestis* in the lungs of inoculated mice by 86% after 24 h of infection. These experiments further demonstrate that predatory bacteria may serve to combat Gram negative bacterial infections, including those considered potential bioweapon agents, in the future.

## 1. Introduction

The United Nations General Assembly met in 2016 to discuss the crisis of antimicrobial resistance (AMR), in the face of predictions that if AMR continues to spread at current rates, there would be an estimated 10 million deaths globally by 2050 [1]. WHO recently released an analysis of the clinical development pipeline, accompanied an urgent call for new treatment for 12 classes of priority pathogens [2]. Extremely drug-resistant strains of *Acinetobacter baumannii, Klebsiella pneumoniae* and *Pseudomonas aeruginosa* are forcing clinicians to turn to antibiotics long abandoned due to toxicity, such as colistin and tigecycline [3]. However, true to form, recent strains of clinical *K. pneumoniae* have demonstrated colistin resistance, injecting a new level of alarm among infectious disease researchers, clinicians and public health practitioners [4]. 

While the creation or discovery of new antibiotics with novel mechanisms of action are one solution, alternative approaches are also required. Bacteriophages have proven to be one possible option and are already in use in several countries [5,6,7]. Another approach is the use of predatory bacteria, a group of Gram negative proteobacteria, including *Bdellovibrio bacteriovorus* and *Micavibrio aeruginosavorus*. These species are obligate predators of Gram-negative bacteria, and have been proposed to be used to treat multidrug-resistant bacterial infections [8,9]. Previous studies from this laboratory with rat and mouse models have shown that the predator bacteria, administered at high levels via lung, tail vein or colon, are inherently non-pathogenic, and are cleared from tissues within 48 h by innate immune mechanisms [10,11,12,13]. Further, it was demonstrated that colony-forming units (CFUs) of *K. pneumoniae* introduced into the lungs of rats can be reduced almost 3 logs by subsequent administration of *B. bacteriovorus* [14]. Other groups have explored chicken and zebrafish models; these studies indicated both safety and efficacy in Gram negative exposures [15,16]. 

The life cycle of the predator bacteria involves attachment to the outer leaf of the outer membrane of the Gram-negative prey cell. *B. bacteriovorus* uses a Type IV-based mechanism to enter the periplasm [17] and, as the Gram-negative rod is converted to a spheroidal “bdelloplast”, begins to deplete nutrients from within the cytoplasm by an as yet undefined mechanism. A second predatory species, *M. aeruginosavorus*, attaches to the outer membrane and remains there, leaching intracellular nutrients from outside the prey cell. Both genera replicate *in situ* and, as nutrient supplies are exhausted, eventually destroy and abandon the prey, releasing progeny which restarts the life cycle by attaching to new prey. 

The host range of predation by the predatory species of the genera *Bdellovibrio* and *Micavibrio* is quite broad among Gram negative species [18]; in addition, virulent strains of bacterial select agents, including *Burkholderia mallei, Francisella tularensis* and *Yersinia pestis*, are susceptible to predation in vitro [19]. *Y. pestis* is the causative agent of plague, which exhibits three major forms of disease: bubonic, septicemic and pneumonic [20,21,22]. Of the three, pneumonic disease, transmitted person-to-person, is the most severe. The course of disease is described as biphasic: early immune response failures during the first 24 h permit rapid replication in the lungs. This inadequate response is followed by a massive anti-inflammatory response that ramps up cytokine expression and host tissue damage, which facilitates further transmission of aerosols [23]. Many of the details of the infection have been worked out in rodent models, and they appear accurately to reflect human disease [20,23,24]. Early constraint of bacterial replication would lead to positive outcomes in host response to infection. Here we examine the ability of *B. bacteriovorus* to reduce CFUs at early timepoints in the lungs of mice exposed to virulent *Y. pestis*. 

## 2. Materials and Methods 

### 2.1. Bacterial Strain and Growth Conditions

The predatory bacterial strain used in the study was *Bdellovibrio bacteriovorus* 109J (ATCC 15143). Predatory bacteria were cultured as described previously [18]. In brief, predator stock-lysates were prepared by co-culturing the predators with host cells in HEPES (4-(2-hydroxyethyl)-1-piperazineethanesulfonic acid) medium supplemented with 3 mM MgCl_2_ and 2 mM CaCl_2_. The co-cultures were incubated at 30 °C on a rotary shaker until the culture cleared. To grow the predators for each predation experiment, 2 mL of predatory bacteria from the stock-lysates were added to 20 mL of HEPES containing 2 mL overnight washed host cells (2 × 10^8^ CFU/mL final concentration). The *B. bacteriovorus* co-culture was incubated for 24 h. Thereafter, the co-cultures were filtered through a 0.45 μm Millex pore-size filter (Millipore, Billerica, MA, USA) to remove residual prey and cell debris (filtered lysate). To further purify and concentrate predator samples, filtered lysate was pelleted three times by centrifugation at 29,000 g for 45 min using a Sorvall LYNX 4000 centrifuge (Thermo Fisher Scientific Inc, Waltham, MA, USA). Each time, the pellet was washed and re-suspended in 50 mL of phosphate buffered saline (PBS). For the last wash, the predator pellet was re-suspended in 1–2 mL of PBS solution to reach a final optical absorbance of ∼0.2 at 600 nm (9.6 × 10^9^ PFUs/mL). To confirm that the samples were free of any contamination, 50 μL aliquots of the predator samples were removed and plated on LB agar and TSB-blood plates. *Yersinia pestis* CO92 (BEI Resources, NR-641) was grown at 37 °C in BBL™ Brain Heart Infusion broth (Becton-Dickenson, 211059, Franklin Lakes, NJ, USA).

### 2.2. Mice

Wild type C57BL/6 (J) mice were purchased from the Jackson Laboratories (Bar Harbor, ME, USA). The mice were housed under pathogen-free conditions at the Rutgers New Jersey Medical School animal facility. All experiments were performed in accordance with the protocols approved by the Institutional Animal Care and Use Committee of Rutgers New Jersey Medical School (protocol #13112A1), the Institutional Biosafety Committee and the Animal Care and Use Review Office of the U.S. Army Medical Research and Material Command. 

### 2.3. Respiratory Inoculation Model

Virulent bacteria (10^5^ *Y. pestis* strain CO92) were introduced by intranasal inoculation of C57BL/6 mice to model a respiratory infection. Animals were lightly anaesthetized with 3% isoflurane in oxygen for four minutes using an isoflurane vaporizer. Twenty-five μL of purified bacterial suspension (1 × 10^5^ CFUs/inoculation) were gently applied at both nostrils. Mice were inoculated with either PBS or *Y. pestis* at the concentrations indicated. At 30 min, 6 h, 12 h and 18 h post inoculation, 25 µL of either PBS or *B. bacteriovorus* (2.4 × 10^8^ PFU/mouse) strain 109J were introduced by nasal inoculation. After initial exposure, animals were observed for the following 24 h and visually assessed for signs of infection, illness and discomfort. Two mice were left untreated. To assess the survival of *Y. pestis* bacteria in the treated and untreated mice, the animals were sacrificed at 24 h post inoculation, lung samples were collected, homogenized and plated for CFU determination. Note that we have performed CFU counts only for the pathogen species, *Y. pestis*; the extent of survival of the predator strain, *B. bacteriovorus*, is usually measured by qRT-PCR, as determining plaque-forming units tends to be highly variable. Please refer to Shatzkes et al. for multiple repeated survival levels in the lungs of rats and mice [10,14]. 

### 2.4. ABSL3 Select Agent Laboratory

All work with select agents was carried out in the Biosafety level three laboratory of the Rutgers-New Jersey Medical School Regional Biocontainment Laboratory, located at the International Center for Public Health and Public Health Research Institute, 225 Warren Street, Newark NJ 07193. Registration number C20170322-1887, Effective date 22 March 2017 and Expiration date 22 March 2020. All protocols were reviewed by the Institutional Biosafety Committee for biosafety, biosecurity and dual use compliance. The implementation of each working protocol was accompanied by a risk assessment and evaluated by an internal protocol committee before execution. 

## 3. Results and Discussion

The spread of antibiotic resistant infectious disease agents is one of the world’s greatest contemporary crises. Over the past several decades, emerging and reemerging infectious diseases have had a grave impact on society and economic stability across the globe. The combination of lives lost (>13 million per year [25]) and the cost of outbreaks (the recent Ebola outbreak approached $53 billion [26]) is exacerbated by the increase in multidrug resistant strains of bacteria, viruses and fungi. While the past two decades of biomedical research have seen a greatly expanded understanding of pathogenesis and immunology, novel antimicrobial development has been slow, and very few new drugs have entered the pipeline [27,28,29].

Predatory bacteria represent an alternative approach to traditional antibiotics, which target essential cellular functions such as protein, DNA, RNA and cell wall synthesis. Predatory bacteria attack and destroy Gram negative bacteria irrespective of growth state or antibiotic resistance status. To date, studies of *K. pneumoniae* [14] and Shigella [15] have demonstrated that the predator bacteria may indeed have some utility in the control of Gram negative infections in animals. 

In addition to the organisms listed above, we explored the possibility of using the predators to combat serious infections caused by potential biological weapons agents. Of the eleven “Tier 1” Select Agents in the US Federal Select Agent Program, five are Gram negative bacteria. In a previous study, we examined the ability of *B. bdellovibrio* to attack and eliminate *Y. pestis*, *Francisella tularensis*, *Burkholderia mallei*, *B. pseudomallei* and *Brucella melitensis* bacteria in liquid culture [19]. Of these, the latter two species were not attacked by *B. bacteriovorus* or *M. aeruginosavorus*. *Y. pestis* strains were robustly attacked by both predator species and reduced in in vitro predation experiments by 50% over 48 h of co-culture. 

The primary objective of our study was to determine whether predatory bacteria are able to reduce the bacterial burden of the causative agent of plague, *Y. pestis*, in an *in vivo* mammalian model of pulmonary plague. We intranasally inoculated C57 Bl/6 mice with virulent *Y. pestis* by inoculating a total of 10^5^ *Y. pestis* bacteria into both nostrils. The animals were then treated with PBS or *B. bacteriovorus* 109J at 30 min and 6, 12, and 18 h post-inoculation. All animals were sacrificed at 24 h. 

Table 1 shows the outline of the experiment and the survival of the animals after 24 h. Previous work from our lab demonstrated that the predator bacteria alone—Even at high levels of inoculum—Cause no ill effects up to as many as 40 days post inoculation by a number of routes of inoculation, including nasal, intravenous and intra-rectal [10,12,13]. Here, all but one of the animals exposed to *Y. pestis* show no symptoms within the first 36 h (see discussion below).

**Figure 1:**
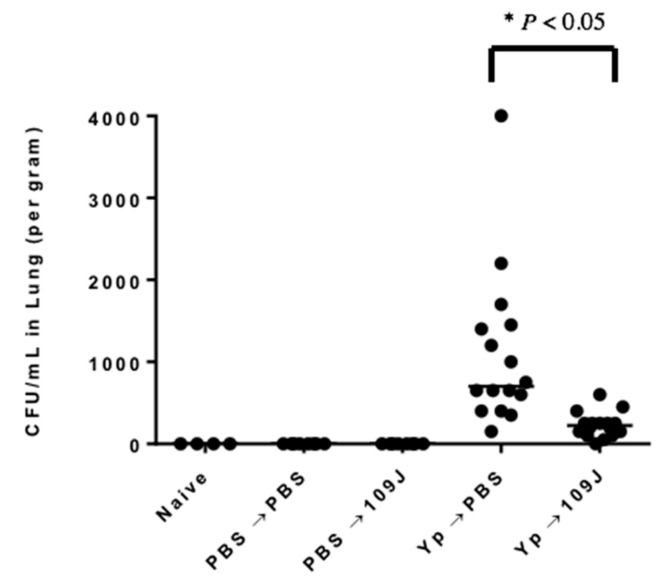
CFU/mL of *Y. pestis* CO92 recovered from entire lung at 24 h post-infection. Animals were exposed to 10^5^ CFU of *Y. pestis* treated with predators at 30 min, 6, 12, and 18 h post-infection (4 doses). *n* = 16 per treatment group. Data presented as median. Significant differences between treatment groups and respective controls were analyzed using Mann-Whitney test (* *p* < 0.05).

Figure 1 show the CFUs recovered from homogenized whole lung tissue of mice. As expected, no colonies of *Y. pestis* were recovered from the lungs of mice in the naïve or PBS-inoculated control groups. In the experimental groups, a median of 7 × 10^2^ CFU/lung tissue was recovered from mice inoculated with 10^5^ CFUs *Y. pestis* CO92. The lungs of all 16 animals contained *Y. pestis* bacteria. In contrast, the lungs of animals exposed to 10^5^ CFU of *Y. pestis* CO92 and then treated with 10^9^ PFUs of *B. bacteriovorus* 109J at 30 min and 6, 12, and 18 h post-inoculation yielded 1 × 10^2^ CFUs/lung. Fourteen of the 15 infected and treated animals contained smaller numbers of *Y. pestis* CFUS and one animal’s lungs were completely cleared of the virulent bacteria. These values represent an 86% reduction of lung CFUs within 24 h of inoculation, based on median values. The data represent a significant, if modest, reduction in CFUs compared with the CFUs recovered from the untreated control group and are the first published data of the use of predatory bacteria to control dissemination of a Select Agent in a mouse model. 

Human pneumonic plague syndrome is closely reflected in the mouse model, with symptoms beginning 2–3 days after exposure, and fatalities appearing at about 72 h. In the mouse, studies have revealed a “biphasic disease” [23]. Within the first 36 h after infection, the lungs of the animals are barely distinguishable from those of uninfected controls: there is rapid bacterial replication in the lung and little to no measurable immune response. The bone marrow is activated and neutrophils flood the bloodstream, but their ingress into the lung is blocked by the pathogen’s interference with the expression of chemoattractants such as KC, MIP-s and G-CSF [23,30]. Then, at 36–48 h, the tide turns: there is sudden increase in pro-inflammatory cytokines and chemokines, neutrophils flood into the lung, and lung tissue destruction with edema and hemorrhage quickly follow. Unless bacterial replication (and accompanying gene expression) is blocked by antibiotic treatment early after exposure, the infection is fatal [23]. Thus, the specific reduction of *Y. pestis* CFUs caused by inoculation of *B. bacteriovorus* 109J after infection would likely lead to interference with the pathogen’s modulation of the host immune response. 

The categorization of *Y. pestis* as a Tier 1 Select Agent by the USG is the result of its use historically as a biological weapon [31,32]. Yet plague has a long and storied past and remains a formidable human pathogen in its own right. The Justinian Plague of 542 and the Black Death of 1346 c.e. were followed by the third pandemic in the late XIX century, killing millions and disrupting societies all over the globe. The pandemic phase of the disease died out but not before establishing an endemic foothold in a number of places, including western United and States and central Madagascar. This island nation off the coast of Africa suffers yearly from seasonal bubonic plague, representing about 400 cases [33]. There was a major outbreak of pneumonia plague between August and November of 2017, resulting in a total of 2417 cases in Madagascar, with 209 deaths (9% case fatality rate) [34]. A recent study of AMR in three unrelated *Y. pestis* strains from two humans and one rat in Madagascar showed unrelated and transmissible plasmids and suggests that AMR can be acquired and transmitted under natural conditions by this bacterium [35], further suggesting that alternative approaches, such as the predatory bacteria described here to combat Gram negative infections, may be in demand in the near future.

## Figures and Tables

**Table 1:** Survival of mice 24 hours post-infection with *Y. pestis*.

Treatment ^1^	# of Mice	% Viable (24 h)
naïve	4	100
PBS → PBS	8	100
PBS → *B. bacteriovorus* 109J	8	100
*Y. pestis* CO92 → PBS	16	100
*Y. pestis* CO92 → *B. bacteriovorus* 109J	16	94 ^2^

^1^ initial inoculum → dosed inoculum. ^2^ probable cause of death: hypothermia during anesthesia.
